# A Systematic Review of Pharmacist-Led Antimicrobial Stewardship Programs in Sub-Saharan Africa

**DOI:** 10.1155/2022/3639943

**Published:** 2022-10-13

**Authors:** Phanice Ajore Otieno, Sue Campbell, Sonny Maley, Tom Obinju Arunga, Mitchel Otieno Okumu

**Affiliations:** ^1^College of Medical, Veterinary and Life Sciences, School of Life Sciences, University of Glasgow, Glasgow, UK; ^2^Department of Health, County Government of Kisumu, PO Box 2738-40100, Kisumu, Kenya; ^3^Department of Health Informatics, Maseno University, Private Bag, Kisumu, Kenya; ^4^Department of Public Health Pharmacology and Toxicology, Faculty of Veterinary Medicine, University of Nairobi, PO Box 29053-00625, Nairobi, Kenya

## Abstract

**Background:**

The misuse of antibiotics contributes significantly to antimicrobial resistance (AMR). Higher treatment costs, longer hospital stays, and clinical failure can all result from AMR. According to projections, Africa and Asia will bear the heaviest burden of AMR-related mortalities in the coming years. Antimicrobial stewardship (AMS) programmes are therefore critical in mitigating the effects of AMR. Pharmacists may play an important role in such programmes, as seen in Europe and North America, but the impact, challenges, and opportunities of pharmacist-led antimicrobial stewardship interventions in Sub-Saharan African hospitals are unknown. The purpose of this systematic review was to assess the impact, challenges, and opportunities of pharmacist-led antimicrobial stewardship interventions in Sub-Saharan African hospitals.

**Methods:**

The Joanna Briggs Institute (JBI) guidelines were used to search for peer-reviewed pharmacist-led studies based in hospitals in Sub-Saharan Africa that were published in English between January 2015 and January 2021. The PubMed, Embase, and Ovid databases were used.

**Results:**

Education and training, audits and feedback, protocol development, and ward rounds were identified as primary components of pharmacist-led antimicrobial stewardship interventions in Sub-Saharan Africa. The pharmacist-led antimicrobial interventions improved adherence to guidelines and reduced inappropriate prescribing, but were hampered by a lack of laboratory and technological support, limited stewardship time, poor documentation, and a lack of guidelines and policies. Funding, mentorship, guidelines, accountability, continuous monitoring, feedback, multidisciplinary engagements, and collaborations were identified as critical in the implementation of pharmacist-led antimicrobial stewardship programmes.

**Conclusions:**

These findings suggest that pharmacists in Sub-Saharan African hospitals can successfully lead antimicrobial stewardship programmes but their implementation is limited by lack of mentorship, accountability, continuous monitoring, feedback, collaborations, and poor funding.

## 1. Introduction

Antibiotics are the most commonly prescribed drugs worldwide [1–4]. According to a study on antibiotic prescribing practice and adherence to guidelines in primary care in South Africa, antibiotics were used inappropriately in 67.9% of public and 60.8% of private hospitals in the region [4]. A systematic review and meta-analysis of antibiotic prescription practices in primary care in low-and middle-income countries (LMICs) established that the antibiotic pooled prevalence proportion ranged from 44% to 60% in LMICs [3]. De Kraker and colleagues reported that antimicrobial resistance was associated with clinical failure, excess morbidity, increased medical costs, and increased length of hospital stay [5, 6]. O'Neil and colleagues project that there may be 10 million global AMR-related deaths by 2050, primarily in LMICs [7]. The attitude, knowledge, and practices of prescribers, limited infrastructure, a lack of reliable clinical diagnostics, insurance reimbursements, financial incentives from medical representatives, and out-of-pocket expenditure are all major contributors to inappropriate prescribing of antibiotics in hospitals [8]. If no action is taken, hard-won gains against major infectious diseases such as tuberculosis (TB), HIV, and malaria in LMICs are likely to be eroded due to inappropriate antimicrobial use.

The World Health Organization (WHO), in collaboration with other professional, national, and international organizations, has advocated for and led the development of frameworks for antimicrobial stewardship programmes (ASPs) [9, 10].

ASPs are a comprehensive set of interventions involving quality improvement activities that, when combined, represent cohesive strategies aimed at ensuring the prudent use and ongoing efficacy of available antimicrobials [9, 10]. Antimicrobial stewardship is achieved by preventing their unnecessary use and providing limited and targeted therapy in situations where these medications are justified [11]. ASPs strive to improve patient outcomes and overall patient care quality [11].

Reviews have shown that ASPs are effective in reducing antibiotic treatment duration, increasing antibiotic policy adherence, reducing resistance, mortalities, morbidities, and antibiotic costs, as well as healthcare-associated infections and prolonged hospitalization [12]. Antimicrobial Stewardship Programs (ASPs) are critical in advancing the Sustainable Development Goals (SDGs) by reducing poverty, economic costs, and increasing productivity [13]. The World Bank estimates that by 2030, 24 million people will be severely affected by poverty. If the problem persists, the economic costs of AMR could rise to 120 trillion dollars by 2050 [13].

A comprehensive stewardship programme should include continuous educational programmes; continuous monitoring and audit of antibiotic consumption patterns with feedback to clinicians; guideline development for infection treatment; antibiotic de-escalation to facilitate transition from broad spectrum to narrow spectrum; antibiotic restriction programmes; antibiotic formulary development; and enforcement of correct drug regimens [14].

The Centers for Disease Control and Prevention (CDC) has classified various types of AMS programmes, including pharmacy-driven programmes that focus on antibiotic dose adjustments; dose optimization; documentation of antibiotic indications; alerts on unnecessary duplicative therapy; changes from intravenous to oral treatment; identification and prevention of antibiotic-related drug interactions; and carrying out automatic stop orders [15].

Sub-Saharan Africa (SSA) is not immune to the growing effects of AMR as a result of inappropriate prescribing. This is due in part to a lack of quality assured microbiology labs, as well as a lack of prioritization of AMR in comparison to other public health concerns [16]. SSA has the highest burden of infectious diseases in the world, with a high prevalence of tuberculosis and HIV, exacerbating high rates of antibiotic use in hospitals. According to the United Nations Program on HIV/AIDS (UNAIDS), the region accounts for 71% of the global HIV burden [17].

The situation is dire, with infections like dysentery and pneumonia no longer treatable with first-line medication [18]. Incidences of resistance in these infections and infectious diseases can result in poor clinical outcomes and increased mortality [18]. Stewardship activities in the region are low and thus there is a need to increase these activities. The most significant barrier observed is a lack of institutional and national support for AMS policies [19].

Infectious disease (ID) pharmacists, who are critical in leading stewardship interventions, are in short supply in SSA [20, 21]. However, recent studies of both public and private healthcare facilities in SSA have shown that, despite the challenges, with adequate training, pharmacists in SSA can develop and direct AMS programmes similar to their counterparts in the United Kingdom (UK) or the United States of America (USA) [20, 21]. Recent reviews have clearly demonstrated the effectiveness of pharmacists leading and playing a central role in stewardship activities by demonstrating a decrease in mortality, length of hospital stay, treatment duration, cost, and antibiotic utilization [22, 23].

However, in contrast to low-income countries, pharmacists' involvement in hospital-based AMS programmes is well established in many high income countries [24]. In high-income countries, the role of pharmacists has expanded and is critical in implementing AMS goals across the continuum of care, including inpatient and outpatient hospital settings [25].

Successful pharmacist-led AMS interventions can amplify the roles pharmacists can play as SSA stewards [26], and many studies have been conducted to assess the impact, effectiveness, and feasibility of pharmacist-led antimicrobial stewardship interventions in both public and private healthcare settings, as well as their clinical outcomes, particularly in high income countries [21, 27].

The first systematic review evaluating the effectiveness of pharmacists leading AMS interventions was conducted in parts of North America and Europe [22]. However, no systematic synthesis of evidence has been conducted to assess the impact of pharmacist-led AMS interventions in enhancing quality of care and promoting judicious use of antimicrobials in hospitals in SSA. The purpose of this study was to assess the impact of pharmacist-led antimicrobial stewardship interventions in hospitals in Sub-Saharan Africa and to identify existing opportunities and challenges that can be leveraged to improve stewardship activities in the region.

## 2. Methods

This systematic review was carried out in accordance with the Preferred Reporting Items for Systematic Review and Meta-Analyses (PRISMA) guidelines [28].

Table 1 shows how the Population, Intervention, Context, Outcomes, and Study Setting (PICOS) framework was used to guide the search strategy and eligibility criteria.

### 2.1. Inclusion Criteria

Pharmacist-led AMS interventions were carried out in SSA region. Pharmacist-led AMS interventions were conducted in both private and public hospital settings and in human population/humans. Quantitative studies focusing on active/practical implementation of pharmacist-led AMS programs in hospitals. Peer-reviewed journals that have been published in English and studies done between January 2015 to September 2021 were included.

### 2.2. Exclusion Criteria

Pharmacist-led AMS interventions were carried outside SSA. Antimicrobial stewardship programs (AMS) in agriculture, animals, and commentaries or expert opinion on AMS; community based AMS initiatives were excluded. Non-English and non-peer-reviewed articles; studies not available in full text and done in non-human health setting; studies that reported on treatment of infections or prevalence of antimicrobial resistance patterns; and pharmacist-led AMS of other classes of drugs (antidiabetics and antihypertensives) other than antimicrobials were excluded.

### 2.3. Sources of Data and Search Strategy

For all relevant articles, a search of published journals was conducted using the databases Embase (Ovid) and PubMed. The search strategy was created with the help of an experienced University librarian on the subject. The search strategy used various techniques to maximize and refine the search. Embase Ovid (2015 to present) was used to map terms like “Antimicrobial stewardship” to the subject (MeSH) heading, which was then followed by a search within the title (“ti”) or abstract (“ab”). Proximity search (“adj3”) was used to count the number of words that were separated from each other in the title or abstract. To narrow the search, truncation (Pharmacist^*∗*^ and Hospital^*∗*^) was used. To broaden and narrow the search, the Boolean operators “OR” and “AND” were used.

To identify any potential studies, a hand search for additional relevant papers using the snowballing technique was conducted. Appendix 1 shows the Embase database search strategy. The following keywords were derived using the PICOS format, and searches were conducted using combined keyword phrases: (Antimicrobial stewardship OR Antibiotic stewardship) AND (pharmacist(s) OR Hospital pharmacist(s) OR Clinical pharmacist(s)) AND (Sub-Saharan African Regions). The search strategy involved combining Sub-Saharan African regions with pharmacists, hospitals, and antibiotic or antimicrobial stewardship terms. PubMed journals were found by searching for “Pharmacist-led AMS.” Filters were also used to narrow down the search. To identify potential papers, snowballing was used.

### 2.4. The Selection Procedure

Endnote reference management software was used to import search results. The literature search results were examined for duplicate identification and removal. Duplicates were removed manually as well as using the endnote software's duplicate function. The remaining articles were screened for titles and abstracts before being assessed for full text based on predefined inclusion/exclusion criteria. The literature on pharmacy-led AMS interventions was found, analyzed, and summarized.

### 2.5. Extraction and Synthesis of Data

The author created a data extraction sheet to extract and synthesize data from selected papers. The following fields were included on the data extraction sheet: author, year of study, country, study location, target population, study design, participants, intervention, study objectives, data collection method, sample, sampling, and sample size; key findings and quality ratings. The validity, reliability, and accuracy of the included studies were determined using the Joanna Briggs Institute (JBI) critical appraisal tool (Critical Appraisal Tools | Joanna Briggs Institute, 2021). In order to report the key findings, a thematic analysis was performed by combining similar observations.

### 2.6. Ethical Considerations

The study did not require ethical approval because it did not involve conducting primary or secondary research that would necessitate the use of patients' data. It did not include any interventions that required the use of human subjects. It entailed compiling evidence from previously published papers.

### 2.7. Data Availability

All data generated or analyzed during this study are available in this manuscript.

## 3. Results

**Figure 1:**
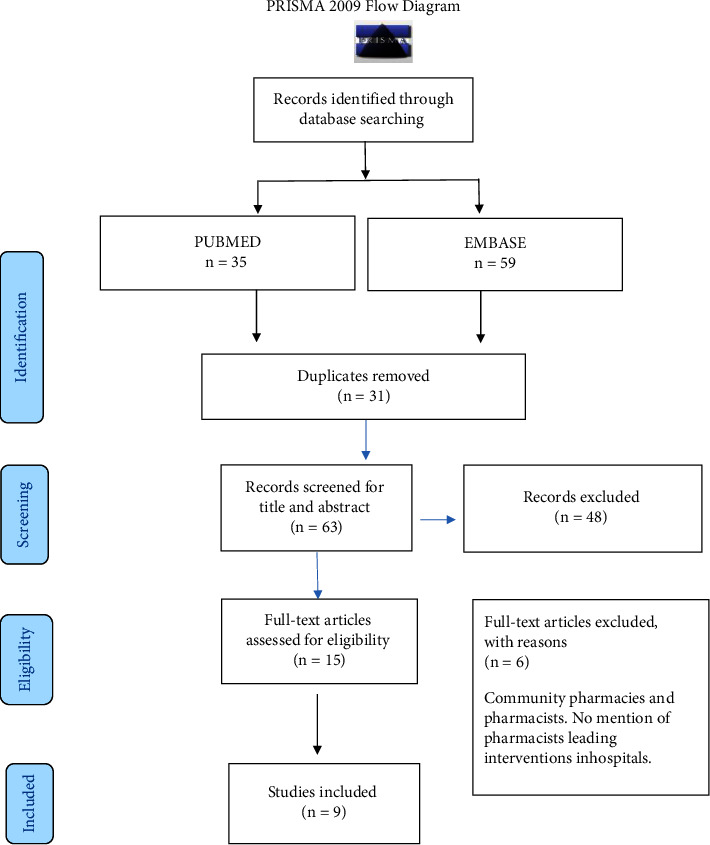
PRISMA diagram literature search process.

The search yielded 94 papers (35 from PubMed and 59 from Embase; Ovid). 31/94 articles were duplicates. Thus, the titles and abstracts of 63/94 of the articles were screened for suitability. From these articles 54/63 were excluded because the studies involved AMS stewardship in community pharmacies, had no mention of the AMS being pharmacist-led, were not in SSA, and did not involve active, practical implementation of pharmacist-led AMS programs, which was this study's focus. Therefore, only nine papers were included in the final review (*I*) (Figure 1).

### 3.1. Included Studies' Characteristics

The Joanna Briggs Institute (JBI) critical appraisal checklist was used to evaluate nine articles. The quality assessment score ranged from six to eight (Table 2). Appendices 2 and 3 contain the full assessment. Following quality control, all nine articles were included. All of the included studies were quantitative in nature and adhered to the positivism paradigm. Four studies were conducted in South Africa [21, 26], one in Ghana [29], one in Ethiopia [30], one in Nigeria [27], and one in Kenya [31]. Eight studies used a quasi-experimental study design, with six studies involving pre and post interventions [26, 27, 29–31]. Two studies used a longitudinal cohort survey (before and after implementation) [21]. Two studies used point prevalence surveys [29, 32]. Five studies were carried out in public hospitals [27, 29–32], three in private hospitals [21], and one in both public and private hospitals [26]. All of the studies used patient medical records to compile data into standardized collection templates/sheets (Microsoft excel).

The convenience sampling method was used in all of the studies. Two studies investigated patients' surgical antibiotic prophylaxis treatments [21, 27]. Two studies examined patients' treatment for a single disease condition, community acquired pneumonia [26, 31]. Three studies investigated how patients were treated for a variety of diseases [21, 30, 32]. Two studies investigated the prevalence of antibiotic prescribing for patients' treatments [29, 32]. All studies were conducted in hospitals, with nonspecialized pharmacists playing key roles in intervention implementation (Table 2). Variations in the quality of included studies were discovered. Because eight studies lacked control groups, it is possible that confounding variables were overlooked. Selection bias could exist as a result of nonrandom assignments. Small sample sizes in two studies may have an impact on the generalizability of the results [29, 31]. Most studies, however, achieved greater validity, reproducibility, and reliability of results because intervention outcomes were replicated in multiple centers with multiple measurements taken and adequate data analysis was used. The use of accurate measurements in accordance with CDC and WHO standards increased reliability [10, 15].

### 3.2. Thematic Synthesis

Thematic synthesis of evidence was performed by consolidating similar observations to establish new themes of evidence. This led to the generation of five distinct themes with subthemes as well (Table 3).

#### 3.2.1. Theme 1: Interventions of Pharmacist-Led AMS

*Sub-Theme 1.1 Education and Training.* Pharmacist led AMS interventions in both public and private hospitals contained an educational component. Education and training were directed to prescribers, healthcare providers, and AMS teams. The same studies have also shown pharmacists providing formal and informal mentorship, education, and training to engage prescribers, other healthcare workers, and multidisciplinary teams in improving antibiotic prescribing and administration in accordance with guidelines and in changing prescribing behaviors. Education and training has been done through face-to-face multidisciplinary workshops, one-on-one training, e-learning resources, use of posters, individual feedback and reminders to prescribers [21, 26, 27, 29–31]. Mentorship sessions were offered to pharmacists by partners to upskill their knowledge [21, 26, 29–31]. Pharmacists were trained on stewardship models that were used to guide implementation [21, 26]. The duration of provision of education varied from one-day training sessions [29] to 33 weeks of extensive learning sessions held intermittently throughout the study [26].

*Sub-Theme 1.2 Audits and Feedback.* Both public and private hospitals employ audits and feedback led by non-specialized pharmacists. This was done by auditing patients' treatments to ensure compliance with antibiotic guidelines. Feedback to clinicians was done verbally, in writing, or through telephone. The feedback was given to clinicians individually or in meetings. This allowed for expeditious concurrent feedback to clinicians and a reduction in the excessive prescription of antibiotics. Audits were done both retrospectively and prospectively to evaluate if there were any improvements in antibiotic management [21, 26, 27, 29–32]. Auditing compliance to diagnostic measures was done by pharmacists in accordance with guidelines [21, 26, 30].

*Sub-Theme 1.3 Development of Protocols.* Guidelines were developed by pharmacists' together with multidisciplinary teams- medical, nursing staff, laboratory staff, and pharmacist- through obtaining consensus and then adapting and modifying the same [27, 29–31]. Apart from developing guidelines, pharmacists disseminated evidence-based practice guidelines to prescribers and other healthcare workers to increase awareness and adherence to treatment guidelines [21, 26].

*Sub-Theme 1.4 Ward Rounds.* Together with other AMS members, pharmacists have been able to assess the appropriateness of antibiotic management for patients on a real-time basis and provide instant oral or written feedback through multidisciplinary rounds and pharmacy audit rounds. Of consideration while carrying out rounds were compliance with antibiotic protocols with regards to redundant therapy or microbiological test results, de-escalation, optimization of antibiotic dosage and intravenous to oral switch. They also reviewed the duration of stop dates of antibiotic treatment in patients restricted dispensing of targeted antibiotics according to approved criteria [21, 26, 27, 30, 31].

#### 3.2.2. Theme 2: Beneficial Impacts of Pharmacist Led AMS Programs

*Sub-Theme 2.1 Improved Adherence to Antibiotic Guidelines.* Several studies reported an improvement in adherence to guidelines including surgical prophylaxis guidelines. Pharmacist-led AMS programs brought about an increase in compliance to local and national standard treatment guidelines [21, 26, 27, 30, 31].

*Sub-Theme 2.2 Reduced Inappropriateness.* Studies documented a reduction in inappropriateness of antibiotic prescribing through pharmacists' interventions. A reduction in the prescribing of third generation cephalosporins for surgical antibiotic prophylaxis from 29.2% before the intervention period to 20.6% after the intervention period was reported [27]. The overall rate of redundant antibiotic prescriptions with pharmacist intervention was reduced from 70.8% before the intervention period to 51.7% after the intervention period [31]. Of 116,662 prescriptions, a pharmacist intervened on 7934 of them. One in every 14.7 prescriptions requires intervention [21]. Discontinuation of unnecessary antibiotics was carried out in 685 prescriptions by pharmacists [30].

*Sub-Theme 2.3 Reduced Antibiotic Utilization.* Pharmacist-led interventions brought about an increase in compliance with treatment protocols. This in turn led to the correct choice of antibiotic being given for the correct condition, at the correct time, the right duration and route of administration, hence a significant reduction in overall utilization of antibiotics [21, 27, 30].

*Sub-Theme 2.4 Reduced Hospitalization.* Reduction in inappropriate prescribing improved patients' outcomes, leading to a reduction in prolonged hospital admissions. Van Den Bergh et al. [26] saw a reduction in hospitalization from eight to six days. A study by Gebretekle et al. [30] revealed that when audits and feedback ceased to be carried out, the length of hospital stay increased significantly by 4.3 days.

*Sub-Theme 2.5 Reduced Healthcare Costs.* With a reduction in antibiotic utilization and reduced hospitalization, studies reported a reduction in healthcare costs by 19% compared to the year preceding the intervention [30]. A reduction by 4.2 dollars per surgical procedure was reported by Abubakar et al. [27].

*Sub-Theme 2.6 Improved Clinical Outcomes.* Compliance with surgical prophylaxis guidelines showed a reduction in surgical site infections (SSI) by 19.7% [21] and a slight decrease in SSIs from 4% to 3.4% [27] with no increases in mortality.

#### 3.2.3. Theme 3: Challenges in the Development of Pharmacist-Led AMS Programs

The challenges were further categorized into two sub-themes; clinical governance related challenges and resources-related challenges.

### 3.3. Sub-Theme Clinical Governance Related Challenges

#### 3.3.1. Lack of Hospital Policies, Guidelines, and Formularies

At the commencement of most of the stewardship programs, lack of availability of guidelines was reported making it difficult for pharmacists to assess patients' antibiotic management. This was seen in both private and public hospitals where antibiotic policies were not in existence and had to be developed at the onset of the pilot project [21, 27, 32]. Pharmacists reported a lack of guideline availability which was a major setback to AMS progress. Guidelines were later developed and provided through mobile platforms [31]. This posed a challenge in guiding rationale use. According to Sneddon et al. [29], adherence to guidelines could not be assessed by pharmacists due to missing guidelines for obstetrics, surgery, and gynecology.

#### 3.3.2. Lack of AMS Multidisciplinary Teams

Studies revealed a lack of AMS multidisciplinary teams at the commencement of the projects. AMS teams were not in existence [21, 29] while in other studies, AMS teams were poorly reconstituted and non-functional with some disciplines not included [21, 31]. Pharmacists could not operate in isolation without the support of well-reconstituted AMS teams.

#### 3.3.3. Poor Documentation

Studies reported the existence of poor documentation of patients' treatment. Brink et al. [21] revealed that the existence of poor documentation on incision time for patients undergoing surgery was the cause of non-adherence in a majority of surgical cases. Poor documentation of antibiotic indications was also seen while pharmacists were conducting surveillance on antimicrobial prescribing patterns [32]. Van Den Bergh et al. [26] reported patients' missing information, making it difficult for pharmacists' to assess patients' antibiotic management. Gebretekle et al. [30] reported a lack of full documentation of clinical diagnosis, antibiotic dosage, and start dates.

#### 3.3.4. Prescribers Knowledge, Attitudes, and Practices

Godman et al. [32], Brink et al. [21], Gebretekle et al. [30], and Sneddon et al. [29] revealed that prescribers had a tendency of initiating empirical antibiotics before taking cultures. Prescribers preferred giving broad-coverage antibiotics “to get it right the first time” without indispensable knowledge of overlapping spectra of activity. They opted for prolonged treatment durations as safer options. One study reported prescribers having lack of awareness of antibiotic policy, having fear of treatment failing with first-line antibiotics, and having low trust in generic medication [31]. Misconceptions in antibiotic use were seen in that prolonged use reduced SSI's [27]. Pharmacists had to employ the interventions mentioned earlier to mentor and guide prescribers to prescribe appropriately.

#### 3.3.5. Guidance and Knowledge on Systems Improvement Methodologies

Van Den Bergh et al. [26] reported a lack of knowledge on system improvement methodologies by pharmacists and other clinical teams. Brink et al. [21] reported gaps in local guidance for the implementation of AMS and improving care. Kerr et al. [31] and Sneddon et al. [29] reported having to train pharmacists on quality improvement methodology employing the Plan, Do, Study, Act (PDSA) cycle and the “Scottish triad approach,” respectively. The Scottish triad approach utilizes education, information, and quality improvement to guide effective implementation of AMS. These methodologies have been seen to be crucial in generating the maximum change in behavior.

### 3.4. Sub-Theme—Resources Related Challenges

#### 3.4.1. Weak Laboratory Infrastructure

Prescribing was without lab investigations which contributed to prescribing empirically and prophylactically. This was a huge setback especially in public hospitals where antibiograms had to be developed with the involvement of pharmacists to guide empiric prescribing. Momanyi et al. [32] documented that 82.6% of total antibiotic encounters were due to empiric prescribing. A lack of availability of microbiological results made it difficult for pharmacists to assess if antibiotic treatment was appropriate [29–32].

#### 3.4.2. Technological Infrastructure

Systems in hospitals were manual, so pharmacists had to collect lab results for patients physically which consumed a lot of stewardship time [29, 30]. Sneddon et al. [29] reported a lack of electronic data collection, therefore prolonging the time taken for data entry by pharmacists. Some studies also reported use of manual systems like paper charts and not electronic physician order entries. There were no electronic dispensing systems [31]. This was a problem, majorly in public hospitals.

#### 3.4.3. Limited Stewardship Time and Inadequate Human Resource

Studies revealed that insufficient time was allocated for stewardship. This was due to staff shortages, competing tasks, and busy work schedules [21, 26, 29]. A study by Brink et al. [21] reported that pharmacists had to discontinue stewardship activities due to competing tasks and the availability of few staff. This problem was seen in both public and private hospitals.

## 4. Theme 4: Enabling Factors for the Development of Pharmacist-Led AMS

### 4.1. Working in Partnerships

Pharmacists relied on working together with partners engaged in the fight against AMR. Kerr et al. [31] revealed that pharmacists partnered with Commonwealth partnerships for AMS programs while in Sneddon et al. [29], they partnered with Scottish Antimicrobial prescribing groups. Gebretekle et al. [30] reported that pharmacists' partnered with Mc Gill partnerships for infectious diseases to support stewardship activities in the various hospitals. Only public hospitals were seen to rely on partnership support.

### 4.2. Mentorship

“Partnerships” enabled shared learning where pharmacists from the UK did work collaboratively with pharmacists in various hospitals in SSA. UK pharmacists supported pharmacists from Ghana, Zambia, Tanzania, and Uganda through mentorship in AMS initiatives [31]. Infectious Disease Pharmacists from the US also supported participating pharmacists from South Africa and the whole multidisciplinary teams in stewardship implementation, offering full guided support with models for implementation [26]. The Scottish Antimicrobial Prescribing Group (SAPG) worked with and mentored lead pharmacists in Ghana to support the development of AMS [29]. In Ethiopia, physician specialized in ID and clinical microbiology, and ID pharmacists from Canada trained pharmacists and gave information sessions for physicians during stewardship implementation [30].

### 4.3. Funding

UK aid funding managed by the Fleming fund provided financial support to two hospitals [29, 31]. A study by Gebretekle et al. [30] reported financial support from the research institute of McGill university Health Centre. Messina et al. [33] received funding support from Ohio university outreach and Van Den Bergh et al. [26] received a grant from Merck. Funding support was majorly seen in public hospitals. Financial support from partners enabled pharmacists to carry out antimicrobial stewardship interventions.

### 4.4. Continuous Professional Development and in Service Training

Continuous professional education of clinical multidisciplinary teams was evident from several studies. Educational meetings by pharmacists were held frequently to increase knowledge and awareness, and further, wall-mounted posters were used as educational reminders to clinicians [21, 26, 27, 30, 31, 33]. Sneddon et al. [29] and Kerr et al. [31] reported that pharmacists' employed a train the trainer model which ensured continuous education took place, thereby improving attitudes and behaviors around antibiotic use.

### 4.5. Continuous Monitoring, Evaluation, and Feedback

Pharmacists carried out continuous audits of antibiotic treatments while giving regular feedback to multidisciplinary teams, with instances of nonadherence addressed immediately. Pharmacists were seen to monitor, document, and collect treatment information for assessment of compliance with antibiotic management policies on a daily and weekly basis [26, 27, 30]. According to Kerr et al. [31], continuous monitoring and evaluation led to the success of stewardship activities by engaging clinical teams over a long period of time with constant feedback. Real-time continuous feedback to front line clinical teams was seen as a pivotal strategy to obtain sustained buy-in [33].

### 4.6. Accountability through Antimicrobial Champion

All studies enhanced stewardship through pharmacists as accountable stewards in driving compliance to the policies and guidelines and ensuring appropriate management of patients. They were champions who promoted and engaged all professional staffs in AMS through employing a number of interventions [21, 26, 27, 29–33].

### 4.7. Multidisciplinary Engagements and Collaboration

Several studies clearly revealed continuous multidisciplinary engagements took place through platforms for shared learning, ward rounds, developing guidelines and antibiograms together [21, 26, 27, 29–31, 33]. In a study by Van Den Bergh et al. [26], pharmacists committed to building strong relationships with multidisciplinary teams, hence providing a platform for shared learning and brainstorming opportunities. Pharmacists were seen as facilitators of engagement among multidisciplinary healthcare service providers.

### 4.8. Development of Guidelines

Guidelines were developed by pharmacists together with a local team of experts which encouraged ownership, easier implementation, and reduced prescribing of certain misused antibiotics. Developing and dissemination of guidelines contributed significantly to compliance with these guidelines [26, 27, 29, 30].

### 4.9. Diagnostic Stewardship

Studies revealed pharmacists' taking part actively in diagnostic stewardship. Pharmacists were seen to palliate some communication deficiencies between the lab and clinicians to optimize management of suspected infections. This enhanced collaboration with microbiology/laboratory departments [21, 26, 30].

## 5. Theme 5: Leadership and Governance

Advocacy for resources and infrastructural support has been determined to be vital for further enhancement and improvement of pharmacist-led AMS in both the private and public health sector.

### 5.1. Financial Resources

Studies revealed the need for national and local leadership to advance partnerships for health so as to improve the quality of healthcare service delivery. With partnerships, financial support was realized [26, 29–31, 33]. Studies also revealed the need for the government to support funding for stewardship programs in order to sustain pharmacist-led interventions. For sustainability, financial support from hospital administration and national and local leadership was deemed to be very crucial [29, 31]. Both external and internal funding were deemed important to advance stewardship activities in major public hospitals.

### 5.2. Human Resources

#### 5.2.1. Employment

Studies brought out the necessity of employment of pharmacy staffs to alleviate staff shortages and allow for dedicated stewardship time. Addressing the gaps in human resources capacity for health was deemed important if pharmacists and other healthcare workers were to be adequately involved in stewardship activities [21, 26, 29].

#### 5.2.2. Training and Mentorship

Several studies have reiterated the importance of investing in the training and development of pharmacists in ID programs and AMS to advance their knowledge and skills [21, 26, 29–31, 33]. Messina et al. reported that AMS should be directed by ID specialists, hence the need for training of pharmacists in ID [33]. These studies revealed a lack of training programs for ID pharmacists; hence, national leaders need to come up with national curriculum to train ID pharmacists.

### 5.3. Guidelines and Protocols for Operations

Studies identified the need for AMS institutional guidelines to guide hospital operations [21, 26, 27, 29–31]. Van Den Bergh et al. [26] advocated the need for national leaders to come up with AMS models for national implementation of specific stewardship programs. These tools have been seen to provide stepwise guidance to pharmacists on the implementation of specific programs [21, 26].

### 5.4. Infrastructural Capacity

Studies revealed the need for improving laboratory infrastructure to advance diagnostic testing and provision of evidence based prescribing practices in hospitals [29–32]. Studies have also shown the need for leadership to improve information and communications technology (ICT) infrastructure in hospitals so as to allow for integrated systems that facilitate smooth delivery of pharmacist-led stewardship activities [30, 31, 33].

## 6. Discussion

This review provides information on the practical implementation of pharmacist-led AMS programmes in SSA hospitals. The following key findings will be discussed: pharmacists' interventions in improving patient care; pharmacists' roles in the implementation of AMS interventions in SSA; the beneficial effects of pharmacist-led AMS on the healthcare system; and the advancement of pharmacist-led AMS in hospitals, with leadership and governance playing a critical role.

### 6.1. Pharmacists' Contributions to Improving Patient Care Quality

Pharmacists used a number of interventions at the same time, including education and training, audits and feedback, protocol development, and ward rounds, which resulted in significant quality improvements in patient care. In isolation, no individual stewardship intervention can be effective [9]. These interventions are consistent with those outlined in the WHO practical toolkit for AMS programmes in LMICs [10]. According to Sneddon et al. [29], clinical teams were trained and pre and posttests were administered to evaluate knowledge gained, with significantly positive results. However, evidence suggests that educational interventions delivered in isolation produce insignificant changes in overall compliance and are mostly ineffective. A study conducted in Australia to reduce inappropriate antibiotic use through a clinician-focused educational AMS programme found no significant change in the pre (18%) and post (15%) intervention periods [34]. When combined with other AMS interventions or bundles, education has been shown to result in improved prescribing behaviours as well as changes in clinician attitudes and practices, as evidenced by our findings [22]. According to studies, the train-the-trainer model is the best way for pharmacists to transfer antibiotic skills to other healthcare workers at scale and at a low cost while ensuring sustainability. Experts train pharmacists to become trainers, who then train other healthcare workers in the train the trainer model. This method promotes ongoing professional development [35]. Continuous professional development requires the delivery of education and training through a variety of information, education, and communication (IEC) materials. The intensity and duration of educational interventions appear to have an effect on their impact and outcomes. According to studies, providers who received education for two years used antibiotics 20% less than those who received single annual education sessions, which used antibiotics 16.5 percent less [36]. AMS education led by pharmacists should be part of ongoing medical and professional development. Prospective audit and feedback intervention provides precise feedback on which antibiotics are prescribed and how they are prescribed [37]. Despite being the most widely used and expensive antimicrobial stewardship strategy, manpower and labor-intensive, this intervention has a higher potential for educational opportunities and change in prescribing practices than preauthorization strategies and formulary restrictions because it is more easily accepted by physicians. Financial sustainability, according to existing publications, is also not an issue [38]. Audits and feedback have been used to identify antibiotic prescribing challenges and to demonstrate the impact of interventions on antibiotic prescribing in terms of de-escalation, duration, timing, antibiotic choice, and culture examinations [39]. Pharmacists have been observed using prescribed antibiotics while providing feedback to physicians on treatment deemed inappropriate [40]. According to a Cochrane review, conducting audits and providing feedback is effective in changing health professionals' behaviour and is a very useful continuous monitoring and evaluation tool for assessing how antibiotics are consumed [37]. Audits and feedback as an active intervention in assessing the improvements taking place are critical for education to be fruitful. When compared to less rigorous education, audit, and feedback, rigorous education, audit, and feedback resulted in a 13% reduction in antibiotic prescribing patterns for respiratory tract infection [36]. Treatment guidelines can help to optimize antibiotic consumption by developing, providing, and disseminating them [41]. Treatment guidelines have been used as a standard to assess antibiotic adequacy [40]. Studies have shown an increase in adherence from 42 percent to 58 percent with pharmacists enhancing adherence to local treatment guidelines and recommendations for infection syndromes and diseases such as community acquired pneumonia, *Clostridium difficile* infections based on facility clinical or national guidelines and local susceptibility data [42]. The development of antimicrobial protocols and guidelines in collaboration with local experts allows clinical teams to take ownership and buy in [43]. It is important to note that, despite clinician-focused education, some guidelines have failed to achieve the desired results in terms of evidence-based measure compliance, owing to limited guidance on how to improve care and a lack of bottom-up approaches to implementation [31]. Working collaboratively with healthcare workers at the hospital level to develop institutional policies and protocols that can be scaled up to higher levels is a bottom-up approach [44]. In order to manage any change process, proper coordination processes and effective teams must be developed. In the United States, the Institute of Medicine's Committee on Quality of Healthcare provides guidance on the coordination of quality improvement change processes, which is critical in improving antibiotic management and building effective teams, according to Kotters' guiding coalition [45]. Models for stewardship implementation have also been identified as critical in facilitating guided implementation and should be based on local context and resources. These models can be embedded in existing systems to increase the adoption of pharmacist-led stewardship programmes [21]. Pharmacy rounds can help you learn about many aspects of antimicrobial prescribing. According to a study conducted in the United Kingdom, nearly 60% of pharmacist contributions are made during multidisciplinary team rounds [46]. Close communication between pharmacists and antimicrobial stewardship teams at the patient's bedside has also been shown in studies to have beneficial effects of stewardship [39]. Incorporating pharmacists into ward rounds is viewed as an effective way to strengthen educational interventions for prescribers. It also makes use of interprofessional shared decision making, in which practitioners can contribute to improving patient care [36]. Several interventions can be made during ward rounds to provide appropriate therapy and improve patient care. These include de-escalation of therapy, dose optimization, intravenous to oral switch, automatic stop orders, redundant therapy, and therapy restriction [10]. All of the above interventions used by pharmacists enable information sharing, improved communication, shared leadership, and shared learning platforms, which are critical for multidisciplinary engagement and collaboration. Clinical teams can brainstorm, provide feedback, and reach a consensus, which is critical in the implementation of stewardship programmes [43].

### 6.2. Pharmacists' Roles in the Implementation of AMS Interventions in SSA

Pharmaceutical care services in Sub-Saharan Africa have been limited to traditional pharmacy practises such as prescription dispensing, medicine procurement, and impromptu compounding [24]. Pharmacists have been underutilized, and their roles in AMS are unclear [47]. On the other hand, developed countries are seeing widespread benefits from expanded roles for pharmacists, which have a direct impact on patient care and the quality of antibiotic drug consumption. The introduction of pharmaceutical care services as a practice-based profession much earlier has been a contributing factor [48]. According to existing literature, pharmacists play an important role in assessing individual patient regimens to optimize therapy, auditing antimicrobial consumption outcomes prospectively and retrospectively, developing and managing antimicrobial guidelines and policies, and providing training and education to clinical teams and patients [48]. Furthermore, they contribute to the development, testing, and implementation of digital AMS platforms, which include electronic prescribing, e-learning, and smartphone apps [49]. They are also important in diagnostic stewardship. As a result, they can serve as a liaison between the microbiological and pharmacy departments [50]. These findings are consistent with our findings, which show that pharmacists are taking on roles in the delivery of stewardship interventions. To advance AMS, it is clear that pharmacists' roles in hospitals in SSA are gradually expanding and should be embedded in routine practice. Pharmacists bring to antimicrobial therapy their unique knowledge of medication use systems, as well as pharmacokinetics, pharmacodynamics, and pharmaco-economic principles, thereby improving patients' clinical outcomes [51]. Because of their positions at the interface between healthcare systems and patients, they are best positioned to aid in the fight against AMR [52]. They are located in the heart of the hospital and have access to patients' medical records, including diagnostic results. They also have up-to-date local formulary information and can thus make informed clinical decisions about antibiotic use in collaboration with multidisciplinary teams and patients, ensuring safe prescribing. Pharmacists are heavily involved in highly effective ASPs programmes, either as programme leaders or as co-leaders [53]. Furthermore, pharmacists trained in infectious disease (ID) are extremely effective in increasing antibiotic consumption, and they frequently assist in the leadership of AMS programmes. In the United States, a study was conducted to compare an AMS programme with an ID pharmacist to programmes that rely on ward pharmacists. Antibiotics were discontinued in 78 percent of cases where ID pharmacists were involved, compared to 33 percent of cases where ward pharmacists were involved. Adherence to local treatment guidelines was also reported to be high when ID pharmacists were involved (96.8 percent vs. 87 percent) [54]. The importance of pharmacists receiving infectious disease training in SSA cannot be overstated. While pharmacists are frequently at the helm of AMS strategies, every member of the healthcare team has a role to play because they cannot carry out health system-wide stewardship plans alone [48]. Multidisciplinary engagements and collaboration with other healthcare professionals such as clinical microbiologists, nurses, doctors, infection control specialists, and most importantly infectious disease physicians are critical, as are well-reconstituted and functional AMS multidisciplinary teams that are well represented to ensure policy translation into action [55]. To improve collaboration among multidisciplinary teams, some hospitals have developed a collaborative practice agreement (CPA) [50]. AMS pharmacists will almost never be successful unless they have the support of the medical staff and a healthy relationship with the ID physician; thus, relationship building and communication are essential for proper coordination and consistent approaches to AMS [56]. In addition to physician leadership accountability, successful stewardship programmes should include drug expertise from a pharmacist leader [53]. In the United Kingdom, AMS programmes are typically led by an Infectious Disease Pharmacist [49]. Pharmacists should be heavily involved in delivering and promoting antimicrobial stewardship programmes in SSA [31]. Many developed countries have seen great success in implementing antimicrobial stewardship (AMS) programmes involving pharmacists [57].

### 6.3. The Benefits of Pharmacist-Led AMS to the Healthcare System

Already, pharmacists have been seen to successfully drive stewardship interventions around the world. In the United States, research on a pharmacist-led culture follow-up programme in the emergency department found a 30% increase in interventions targeting inappropriate antimicrobial prescribing compared to the preceding nursing management [58]. Reviews conducted in parts of Europe and America have shown that pharmacists' leading stewardship interventions resulted in a reduced duration of antimicrobial therapy ranging from 1.0–1.7 days without an increase in mortality [22]. Pharmacist-led interventions have been linked to a 10% reduction in mortality in regions such as China and Brazil, according to published literature [59]. In the United Arab Emirates, reviews conducted with the assistance of a pharmacist revealed significant improvements in guideline-concordant antibiotic selection (80.2 percent), duration of therapy (86.2 percent), and dose (86.2 percent) [23]. Furthermore, it has been observed that physicians rely heavily on pharmacists' antibiotic stewardship recommendations and expertise [46]. According to studies, AMS with a pharmacist resulted in lower antimicrobial consumption than AMS without a pharmacist [23]. Meta-analysis studies conducted in the United Arab Emirates clearly reported lower inappropriateness in antibiotic use with pharmacist involvement (11.88 percent) compared to without (33.7 percent) [23]. When the number of inappropriately prescribed medicines is reduced, the cost of each individual medicine is reduced [60]. In China and the Netherlands, pharmacist-led AMS programmes have been linked to savings of up to 80% and more than 25% on antibiotic prophylaxis per procedure, respectively [46]. As seen in a meta-analysis by Mahmood et al. [23]and a review by Nathwani et al. [61] focusing on North America and Europe, AMS with pharmacist has a positive impact by reducing length of hospital stay by 85 percent, confirming the capability of pharmacist-led AMS to reduce length of hospital stay. There is mounting evidence from both global and analysed studies that pharmacist-led AMS interventions in SSA result in significantly positive clinical and economic outcomes. Such positive outcomes can be used to justify investment in antimicrobial stewardship expansion and infrastructure in SSA. Given the successful studies documented and the global movement in harnessing pharmacists' expertise in delivering stewardship interventions, the capabilities of pharmacists taking on stewardship roles in SSA should be fully utilized [55].

### 6.4. Development of Pharmacist-Led AMS in Hospitals in SSA

It is critical that pharmacists receive specialized training in AMS and infectious diseases. Stewardship programmes in the United States and the United Kingdom have advanced as a result of training investments. A survey conducted in the United Kingdom in 2011 revealed a 1.5-fold increase in the number of specialist antimicrobial pharmacists since the previous survey in 2005. Each hospital surveyed had at least one specialist antimicrobial pharmacist, and 16% of those 16 percent worked full-time in their stewardship roles [62]. The incorporation of the AMS curriculum into undergraduate pharmacy degree programmes should be strongly considered in order to better prepare pharmacists for their roles in hospital stewardship and provide them with a foundation to build on [27, 46, 50]. Recently, there has been a global health leadership training for UK infection pharmacists collaborating with pharmacists in Africa to lead on AMS [31, 63].

### 6.5. Pharmacists Have the Potential to be Antimicrobial Champions

They are drug experts who can effectively provide training and education while also ensuring accountability in AMS through continuous antibiotic monitoring and evaluation. They can be points of contact for implementation, so improving their knowledge of ID and AMS cannot be overstated [64]. Implementation of AMS, particularly in private institutions, has made use of quality improvement methodologies such as Plan, Do, Study, Act (PDSA). Quality Improvement methodology is a well-known iterative and systematic approach to achieving maximum behaviour change [31, 33]. It is important to note that pharmacists' skills beyond infectious diseases are critical for initiating and maintaining AMS programmes [21]. Aside from integrating pharmacy teams in the delivery of AMS interventions, infrastructure support has been identified as critical to enhancing positive outcomes [65]. According to reviews, improved diagnostic testing would be very valuable, and this can undoubtedly be realized by developing more robust systems that use modern technology to support AMS-prescribing principles in a more integrated, user-friendly, and practical manner. Speed of diagnostic testing is a critical factor in effective and efficient pharmacist-led AMS in hospitals, as it has led to long turnaround times in patient results and encouraged empirical prescribing [66]. Laboratory personnel can be effectively trained to provide guidance on the flow of results and the proper use of tests. Creating antibiograms in health care facilities with the assistance of pharmacists can help optimise empiric antibiotic prescribing, especially in resource-constrained settings [10]. It is critical to strengthen ICT in order to integrate stewardship protocols into existing workflow. This can be accomplished by embedding protocols and information at the point of care, facilitating antimicrobial use data collection and reporting, and implementing clinical decision support for antimicrobial use. This will help to alleviate the problem of inadequate documentation [67]. While there is no agreement on staffing recommendations, hospitals with existing programmes recommend that at least 10 hours of clinical pharmacist time per week be dedicated to AMS for every 100 patient beds [68]. However, it has been proposed that institutions use WHO AMS guidelines to help them with staffing and other stewardship requirements [10].

#### 6.5.1. Roles of Leadership and Governance in the Advancement of AMS in SSA

AMS should be prioritized by national, local, and institutional leaders. Leadership dedication and proper AMS governance structures are regarded as critical [67]. According to the CDC, leadership commitment entails allocating the necessary financial, human, and information technology resources to support AMS activities [15]. To support the fundamental role of non-specialized pharmacists in recruiting multidisciplinary teams, coordinating interdisciplinary clinicians, and engaging nurses, leadership commitment from the government, hospital, and clinical sectors is required [69]. The most significant challenge is that most SSA countries' health leaders have failed to prioritize AMR and AMS over other public health concerns, resulting in slow progress in stewardship activities adoption [16]. It is important to note that leadership plays an important role in oversight, accountability, and system design in ensuring the success of AMS implementation [66]. Furthermore, leadership can play a critical role in addressing hospital-specific barriers such as resistance among specific cadres or departments, as well as challenges to clinical autonomy [68]. Both institutional leaders and middle-level managers are included. Dedicated funding or budget is essential to increase pharmacist involvement in ASP because funds are required to train existing pharmacists in ID and AMS as well as recruit more pharmacists. In the United Kingdom, increased funding for AMS development has greatly increased pharmacist participation in ASPs such as antimicrobial stewardship education, surveillance and monitoring of antibiotic utilization, and revision and development of formulary and guidelines [69]. Support from hospital administration, as well as dedicated and ongoing funding, are independent predictors of effective pharmacist-led antimicrobial stewardship programmes. As a result, hospital administrators and public health agencies in Sub-Saharan Africa should be encouraged to provide pharmacists with the necessary support to implement antimicrobial stewardship programmes [70].

Internal and external funding are both critical for advancing AMS activities. However, while strengthening partnerships for health is important in improving healthcare delivery to patients in developing countries, including SSA, dedicated funding and strengthening local capacities by various Ministries of Health (MoH) are critical for sustainability, continuity, and less reliance on donor/partner funding in SSA stewardship activities. Furthermore, most donors and partners are reorienting their strategies and partnership models by collaborating with governments in developing countries to help them achieve more stable, resilient development outcomes and locally sustained results, as well as assisting countries in their “Journey to Self-Reliance.” There should be a shift from top-down to bottom-up approaches in policy formulation, planning, and implementation to allow for embedded improvement initiatives in routine practice, which is critical for achieving long-term benefit in pharmacist-led AMS [68]. Bottom-up approaches to stewardship implementation have been seen to be critical in enhancing ownership and sustainability, with key stakeholders involved in developing AMS policies, a practice that should supersede top-down approaches to stewardship implementation. Bottom-up approaches are important because they allow for stewardship to be aligned with the local context and work with available resources [21]. AMS governance structures in hospitals are critical to the sustainability of pharmacist-led AMS programmes because they provide the necessary authority, oversight structures, and decision-making chain. To advance pharmacist-led AMS programmes, leadership commitment is required; that is, a formal statement or policy indicating that the institution or health care facility supports efforts to improve, monitor, and promote antimicrobial stewardship. Formal statements carry far more weight with hospital personnel than informal communications such as e-mails or newsletters. Increased uptake of AMS implementation in hospitals may not be realized without leadership commitment and support.

### 6.6. The Study's Limitations and Strengths

Randomized control trials (RCTs) are ranked first after meta-analysis and systematic reviews, and they are widely regarded as having the highest level of credibility when it comes to determining causality [21]. RCTs are ideal for studies analyzing evidence in terms of a hierarchy of evidence; however, use of this design may not be ethically appropriate for studies that have shown benefits over time [71]. In contrast, quasiexperimental designs with pre and posttest interventions can be used to determine the impact of healthcare interventions and have been widely used in healthcare studies to improve the quality of medical services [72].

As a result, the majority of studies used nonrandomized experimental studies with pretest-posttest interventional studies, which are ideal and best suited because depriving patients of beneficial treatment or intervention with known efficacy is unethical. It is important to note that quantitative research approaches generally allow researchers to establish causality between interventions and outcomes as well as associations between variables [73], and all of the studies examined were quantitative in nature. However, the lack of control groups may make it difficult to account for confounding variables, limiting the findings' generalizability and transferability. Many antimicrobial stewardship studies have enormous variability, which can limit interpretation and lead to confounding variables, making it difficult to determine cause and effect causal relationships [30]. Despite this, most studies took some of the confounding variables into account when determining cause and effect, increasing the internal validity of the results. Selection bias could occur as a result of non-random assignment, as seen in one study [30], limiting the study's ability to conclude a causal relationship between intervention and outcome and reducing the internal validity of findings. Despite the fact that three of the included studies were single-center studies, which could potentially affect the generalizability of findings, the greatest strength seen in the majority of the studies was that they were multicenter studies, all of which resulted in beneficial outcomes, which could increase the chances of transferability, generalizability, reliability, and credibility of findings to resource-limited settings. Small sample sizes in two studies may limit the generalizability of findings to entire populations. However, nonrandomized experimental studies, according to Harris et al. [71], are a good way to clearly display effects of intervention versus nonintervention and can also help effects of independent variables stand out even with fewer participants. A large data set with measurement criteria based on CDC and WHO standards for process and outcome measures increased the credibility and reliability of the findings [10]. Articles that were not published in English and were not available in full text were excluded, and thus some information may have been missed. The generalizability of results to the entire SSA region may be limited due to the majority of studies being conducted in South Africa, which has been seen to be making great strides in pharmacist-led AMS in SSA when compared to other countries.

In addition, there is a scarcity of data on pharmacist-led AMS studies in the region. However, with the reproducibility of positive beneficial results seen in the majority of studies conducted in multiple centers, finding transferability is very much possible. The majority of the studies also used adequate data analysis with multiple measurements and were of high methodological rigor, increasing the likelihood of results being transferable. These studies can serve as a guide and motivation to pharmacists and healthcare leaders interested in implementing pharmacist-led AMS programmes in Sub-Saharan Africa.

## 7. Conclusions and Recommendations

These findings suggest that pharmacist-led AMS interventions, protocol development, ward rounds, audits, and feedback were effective in increasing adherence to protocols and guidelines, decreasing antimicrobial therapy, and decreasing healthcare costs. This study identified a lack of guidelines, poor prescriber attitudes and practices, lack of AMS teams, laboratory and technological infrastructure, limited stewardship time, and inadequate human resources as major challenges in pharmacist-led AMS programs. Partnerships that resulted in funding and mentorship support were key enablers, as were increased accountability, multidisciplinary engagements, the development of guidelines, diagnostic stewardship, continuous professional development, and monitoring and evaluation with feedback.

Training of pharmacists on AMS interventions, fostering synergies with relevant healthcare stakeholders, resource mobilization, and continuous quality improvement are necessary to support the implementation of pharmacist-led AMS programmes in sub-Saharan hospitals.

## Figures and Tables

**Table 1:** PICOS framework.

Criteria	Determinants
Population	Patients in both public and private sector hospitals.
Intervention	Evidence of pharmacist-led antimicrobial stewardship interventions in hospitals in private and public sectors.
Context	Antimicrobial/antibiotic stewardship in sub-Saharan Africa.
Outcomes	Challenges, advantages, benefits of active implementation of pharmacist-led antimicrobial stewardship interventions in enhancing the quality of patient care and promoting judicious use of antimicrobials/Antibiotics.
Study setting	Sub-Saharan Africa.

**Table 2:** Characteristics of the studies included.

Author/year/country	Objectives	Study design	Target population	Participants	Sample	Sampling and sample size	Data collection method	Quality rating (JBI)
Brink et al., 2016 [21], South Africa	Implementation of pharmacist-led audit and feedback for peri-operative antibiotic prophylaxis.	Pre-post implementation study	34 private hospitals	42 nonspecialized pharmacists. Anesthetists, surgeons, infection prevention practitioners, nurses, theatre managers.	Patients on peri-operative antibiotic prophylaxis	Convenience sampling *N* = 24,206 surgical cases	Patient medical Records, standardized templates using microsoft excel.	7
Kerr et al., 2021 [31], Ghana, Uganda, Zambia, and Tanzania.	Assess compliance with antibiotic policy prescriptions issued to ambulatory patients with moderate or mild pneumonia.	Pre-post study.	Ghana-1 public municipal hospital.	45 pharmacists, AMS teams.	Patients prescriptions	Convenience sampling *N* = 757 prescriptions.	Patients medical records from databases onto excel spreadsheet	6
Gebretekle et al., 2020 [30], Ethiopia.	Assess impact and feasibility of a pharmacist driven intervention on antibiotic utilization.	Single prospective quasi experimental study.	1 teaching and referral hospital.	80 nonspecialized pharmacists (4 clinical pharmacists) AMS teams.	Prescriptions for in-patients receiving systemic antibiotics	Convenience sampling *N* = 1264 (intervention phase), *N* = 1141 (post intervention)	Patients medical records. Structured forms attached to patient charts	7
Momanyi et al., 2019, Kenya. [32]	Determine prescribing patterns of antibiotics in one of the referral hospitals in Kenya	Point prevalence cross-sectional survey	1 referral hospital	Pharmacists	In-patients on systematic antibiotics.	Convenience sampling *N* = 179	Patient medical records, PPS forms, microsoft excel.	8
Brink et al., 2016 [21], South Africa	Assess the reduction of overall antibiotic utilization in hospitals in South Africa through antimicrobial stewardship implementation strategy that utilizes existing resources.	Longitudinal cohort survey. (pre-post)	47 private hospitals	64 nonspecialized pharmacists. Doctors, nursing, clinical staff and infection prevention practitioner.	Patients on antibiotic treatment	Convenience sampling *N* = 116,662	Patients medical records, standardized templates	8
Messina et al., 2015 [33], South Africa.	Evaluate change in adherence with administration of antimicrobials within an hour of prescription.	Prospective multicenter quasi-experimental (pre-post)	33 private hospitals	Nonspecialized pharmacists	Patients receiving intravenous antibiotics.	Convenience sampling *N* = 32,985	Patient medical records	6
Van Den Bergh et al., 2020 [26], South Africa.	Assess the utilization of non-specialized pharmacists in implementing community-acquired pneumonia.	Multicenter prospective cohort study.	39 public and private hospitals	63 nonspecialized pharmacists physicians and other multidisciplinary teams	Adult patients admitted with community acquired pneumonia.	Convenience sampling *N* = 2464	Standardized daily sheets	8
Abubakar et al., 2019 [27], Nigeria.	Evaluate impact of antibiotic stewardship interventions on prescribing of surgical antibiotic prophylaxis.	Prospective pre-post intervention study	2 public tertiary hospitals	Nonspecialized pharmacists.	Obstetric and gynecological surgical cases.	Convenience sampling 226-pre, 238-post	Patients medical records	7
Sneddon et al., 2020 [29], Ghana	Capacity building staffs through training and collection of data on antibiotic use.	Pre-post study	1 public hospital	Nonspecialized pharmacist Medical, nursing team	60 healthcare workers	Convenience sampling	Questionnaires Patients medical records	7

**Table 3:** Key themes and sub-themes.

Key theme	Theme 1: Interventions of pharmacist led AMS.	Theme 2: Impacts of pharmacist led AMS.	Theme 3: Challenges in implementation of pharmacist led AMS.	Theme 4: Enabling factors for implementation of pharmacist led AMS.	Theme 5: Leadership and governance.

Sub-theme	1.1 Education and training. 1.2 Audits and feedback. 1.3 Development of protocols. 1.4 Ward rounds.	2.1 Improved adherence to antibiotic guidelines. 2.2 Reduced inappropriateness. 2.3 Decreased antibiotic utilization. 2.4 Decreased hospitalization. 2.5 Decreased healthcare costs. 2.6 Improved clinical outcomes.	3.1 Clinical governance related challenges 3.1.1 Lack of hospital policies and guidelines. 3.1.2 Lack of AMS multidisciplinary teams. 3.1.3 Poor documentation. 3.1.4 Prescribers attitudes, knowledge and practices. 3.1.5 Guidance and knowledge on system improvement methodologies. 3.2 Resources related challenges. 3.2.1 Weak laboratory infrastructure. 3.2.2 Technological infrastructure. 3.2.3 Limited stewardship time and inadequate human resource.	4.1 Working in partnerships. 4.2 Mentorship. 4.3 Funding. 4.4 Continuous professional development and in service training. 4.5 Continuous monitoring, evaluation and feedback. 4.6 Accountability 4.7 Multidisciplinary engagement and collaboration. 4.8 Development of guidelines. 4.9 Diagnostic stewardship.	5.1 Financial resources. 5.2 Human resources. 5.2.1 Employment. 5.2.2 Training and mentorship. 5.3 Guidelines and protocols for operations. 5.4 Infrastructural capacity.

## Data Availability

All data generated or analyzed during this study are included in the text.

## References

[1] Klein E. Y., Van Boeckel T. P., Martinez E. M. (2018). Global increase and geographic convergence in antibiotic consumption between 2000 and 2015. *Proceedings of the National Academy of Sciences of the United States of America*.

[2] Okoth C., Opanga S., Okalebo F., Oluka M., Baker Kurdi A., Godman B. (2018). Point prevalence survey of antibiotic use and resistance at a referral hospital in Kenya: findings and implications. *Hospital Practice*.

[3] Sulis G., Adam P., Nafade V. (2020). Antibiotic prescription practices in primary care in low-and middle-income countries: a systematic review and meta-analysis. *PLoS Medicine*.

[4] Gasson J., Blockman M., Willems B. (2018). Antibiotic prescribing practice and adherence to guidelines in primary care in the Cape Town metro district, South Africa. *South African Medical Journal*.

[5] De Kraker M. E. A., Wolkewitz M., Davey P. G. (2011). Burden of antimicrobial resistance in European hospitals: excess mortality and length of hospital stay associated with bloodstream infections due to *Escherichia coli* resistant to third-generation cephalosporins. *Journal of Antimicrobial Chemotherapy*.

[6] De Kraker M. E. A., Davey P. G., Grundmann H., Group B. S. (2011). Mortality and hospital stay associated with resistant *Staphylococcus aureus* and *Escherichia coli* bacteremia: estimating the burden of antibiotic resistance in Europe. *PLoS Medicine*.

[7] O’Neill J. (2016). *Tackling Drug-Resistant Infections Globally: Final Report and Recommendations*.

[8] Mohamadloo A., Ramezankhani A., Zarein-Dolab S., Salamzadeh J., Mohamadloo F. (2017). A systematic review of main factors leading to irrational prescription of medicine. *Iranian Journal of Psychiatry and Behavioral Sciences*.

[9] Barlam T. F., Cosgrove S. E., Abbo L. M. (2016). Implementing an antibiotic stewardship program: guidelines by the infectious diseases society of America and the society for healthcare epidemiology of America. *Clinical Infectious Diseases*.

[10] World Health Organization (2019). *Antimicrobial Stewardship Programmes in Health-Care Facilities in Low-And Middle-Income Countries: A WHO Practical Toolkit*.

[11] Charani E., Holmes A. H. (2013). Antimicrobial stewardship programmes: the need for wider engagement. *BMJ Quality and Safety*.

[12] Davey P., Brown E., Charani E. (2013). Interventions to improve antibiotic prescribing practices for hospital inpatients. *Cochrane Database of Systematic Reviews*.

[13] World Bank (2017). *Drug-resistant Infections: A Threat to Our Economic Future*.

[14] Aryee A., Price N. (2015). Antimicrobial stewardship—can we afford to do without it?. *British Journal of Clinical Pharmacology*.

[15] C for DC and Prevention (2017). *Core Elements of Hospital Antibiotic Stewardship Programs*.

[16] Leopold S. J., van Leth F., Tarekegn H., Schultsz C. (2014). Antimicrobial drug resistance among clinically relevant bacterial isolates in sub-Saharan Africa: a systematic review. *Journal of Antimicrobial Chemotherapy*.

[17] Kharsany A. B. M., Karim Q. A. (2016). HIV infection and AIDS in sub-Saharan Africa: current status, challenges and opportunities. *The Open AIDS Journal*.

[18] Porter G. J., Owens S., Breckons M. (2021). A systematic review of qualitative literature on antimicrobial stewardship in Sub-Saharan Africa. *Global Health Research and Policy*.

[19] Howard P., Pulcini C., Levy Hara G. (2015). An international cross-sectional survey of antimicrobial stewardship programmes in hospitals. *Journal of Antimicrobial Chemotherapy*.

[20] Mathew P., Ranjalkar J., Chandy S. J. (2020). Challenges in implementing antimicrobial stewardship programmes at secondary level hospitals in India: an exploratory study. *Frontiers in Public Health*.

[21] Brink A. J., Messina A. P., Feldman C. (2016). Antimicrobial stewardship across 47 South African hospitals: an implementation study. *The Lancet Infectious Diseases*.

[22] Monmaturapoj T., Scott J., Smith P., Abutheraa N., Watson M. C. (2021). Pharmacist-led education-based antimicrobial stewardship interventions and their effect on antimicrobial use in hospital inpatients: a systematic review and narrative synthesis. *Journal of Hospital Infection*.

[23] Mahmood R. K., Gillani S. W., Saeed M. W., Vippadapu P., Alzaabi M. J. M. A. (2021). Impact of pharmacist-led services on antimicrobial stewardship programs: a meta-analysis on clinical outcomes. *Journal of Pharmaceutical Health Services Research*.

[24] Sakeena M. H. F., Bennett A. A., McLachlan A. J. (2018). Enhancing pharmacists’ role in developing countries to overcome the challenge of antimicrobial resistance: a narrative review. *Antimicrobial Resistance and Infection Control*.

[25] Parente D. M., Morton J. (2018). Role of the pharmacist in antimicrobial stewardship. *Medical Clinics of North America*.

[26] Van Den Bergh D., Messina A. P., Goff D. A. (2020). A pharmacist-led prospective antibiotic stewardship intervention improves compliance to community-acquired pneumonia guidelines in 39 public and private hospitals across South Africa. *International Journal of Antimicrobial Agents*.

[27] Abubakar U., Syed Sulaiman S. A., Adesiyun A. G. (2019). Impact of pharmacist-led antibiotic stewardship interventions on compliance with surgical antibiotic prophylaxis in obstetric and gynecologic surgeries in Nigeria. *PLoS One*.

[28] Moher D., Liberati A., Tetzlaff J., Altman D. G., The PRISMA Group (2009). Preferred reporting items for systematic reviews and meta-analyses: the PRISMA statement. *Annals of Internal Medicine*.

[29] Sneddon J., Afriyie D., Sefah I. (2020). Developing a sustainable antimicrobial stewardship (Ams) programme in Ghana: replicating the scottish triad model of information, education and quality improvement. *Antibiotics*.

[30] Gebretekle G. B., Haile Mariam D., Abebe Taye W. (2020). Half of prescribed antibiotics are not needed: a pharmacist-led antimicrobial stewardship intervention and clinical outcomes in a referral hospital in Ethiopia. *Frontiers in Public Health*.

[31] Kerr F., Sefah I. A., Essah D. O. (2021). Practical pharmacist-led interventions to improve antimicrobial stewardship in Ghana, Tanzania, Uganda and Zambia. *Pharmacy*.

[32] Momanyi L., Godman B., Opanga S., Nyamu D., Oluka M., Kurdi A. (2019). Antibiotic prescribing patterns at a leading referral hospital in Kenya: a point prevalence survey. *Journal of Research in Pharmacy Practice*.

[33] Messina A. P., Van Den Bergh D., Goff D. A. (2015). Antimicrobial stewardship with pharmacist intervention improves timeliness of antimicrobials across thirty-three hospitals in South Africa. *Infectious Disease and Therapy*.

[34] Knox M. C., Edye M. (2016). Educational antimicrobial stewardship intervention ineffective in changing surgical prophylactic antibiotic prescribing. *Surgical Infections*.

[35] Goff D. A., Bauer K. A., Brink A. (2020). International train the trainer antibiotic stewardship program for pharmacists: implementation, sustainability, and outcomes. *Journal of the American College of Clinical Pharmacy*.

[36] Satterfield J., Miesner A. R., Percival K. M. (2020). The role of education in antimicrobial stewardship. *Journal of Hospital Infection*.

[37] Carter M., Abutheraa N., Ivers N. (2021). A systematic review of pharmacist-led audit and feedback interventions to influence prescribing behaviour in general practice settings. *International Journal of Pharmacy Practice*.

[38] Chung G. W., Wu J. E., Yeo C. L., Chan D., Hsu L. Y. (2013). Antimicrobial stewardship: a review of prospective audit and feedback systems and an objective evaluation of outcomes. *Virulence*.

[39] Nakamura S., Arima T., Tashiro R. (2021). Impact of an antimicrobial stewardship in a 126-bed community hospital with close communication between pharmacists working on post-prescription audit, ward pharmacists, and the antimicrobial stewardship team. *Journal of Pharmaceutical Health Care and Sciences*.

[40] Apisarnthanarak A., Lapcharoen P., Vanichkul P., Srisaeng-Ngoen T., Mundy L. M. (2015). Design and analysis of a pharmacist-enhanced antimicrobial stewardship program in Thailand. *American Journal of Infection Control*.

[41] Phillips C. J., Gordon D. L. (2015). Pharmacist-led implementation of a vancomycin guideline across medical and surgical units: impact on clinical behavior and therapeutic drug monitoring outcomes. *Integrated Pharmacy Research and Practice*.

[42] Bishop P. A., Isache C., McCarter Y. S., Smotherman C., Gautam S., Jankowski C. A. (2020). Clinical impact of a pharmacist-led antimicrobial stewardship initiative evaluating patients with Clostridioides difficile colitis. *Journal of Investigative Medicine*.

[43] Mack M. R., Rohde J. M., Jacobsen D. (2016). Engaging hospitalists in antimicrobial stewardship: lessons from a multihospital collaborative. *Journal of Hospital Medicine*.

[44] Rayner S. (2010). How to eat an elephant: a bottom-up approach to climate policy. *Climate Policy*.

[45] Baker A. (2001). *Crossing the Quality Chasm: A New Health System for the 21st Century*.

[46] Kader Mohiuddin A. (2019). Pharmacist-led antimicrobial stewardship. *ACTA Scientific Medical Sciences*.

[47] Sharma S., Bowman C., Alladin-Karan B., Singh N. (2016). Antibiotic prescribing patterns in the pediatric emergency department at Georgetown public hospital corporation: a retrospective chart review. *BMC Infectious Diseases*.

[48] Liaskou M., Duggan C., Joynes R., Rosado H. (2018). Pharmacy’s role in antimicrobial resistance and stewardship. *Clinical Pharmacy*.

[49] Gilchrist M., Wade P., Ashiru-Oredope D. (2015). Antimicrobial stewardship from policy to practice: experiences from UK antimicrobial pharmacists. *Infectious Disease and Therapy*.

[50] Fay L. N., Wolf L. M., Brandt K. L. (2019). Pharmacist-led antimicrobial stewardship program in an urgent care setting. *American Journal of Health-System Pharmacy*.

[51] Cipolle R. J., Strand L. M., Morley P. C. (2012). *Pharmaceutical Care Practice: The Patient-Centered Approach to Medication Management*.

[52] World Health Organization (2014). Antimicrobial resistance. https://www.who.int/news-room/fact-sheets/detail/antimicrobial-resistance.

[53] Heil E. L., Kuti J. L., Bearden D. T., Gallagher J. C. (2016). The essential role of pharmacists in antimicrobial stewardship. *Infection Control & Hospital Epidemiology*.

[54] Bessesen M. T., Ma A., Clegg D. (2015). Antimicrobial stewardship programs: comparison of a program with infectious diseases pharmacist support to a program with a geographic pharmacist staffing model. *Hospital Pharmacy*.

[55] Nampoothiri V., Sudhir A. S., Joseph M. V. (2021). Mapping the implementation of a clinical pharmacist-driven antimicrobial stewardship programme at a tertiary care centre in South India. *Antibiotics*.

[56] Gallagher J. C., Justo J. A., Chahine E. B. (2018). Preventing the post-antibiotic era by training future pharmacists as antimicrobial stewards. *American Journal of Pharmaceutical Education*.

[57] Goff D. A., Kullar R., Goldstein E. J. C. (2017). A global call from five countries to collaborate in antibiotic stewardship: united we succeed, divided we might fail. *The Lancet Infectious Diseases*.

[58] Davis L. C., Covey R. B., Weston J. S., Hu B. B. Y., Laine G. A. (2016). Pharmacist-driven antimicrobial optimization in the emergency department. *American Journal of Health-System Pharmacy*.

[59] Li Z., Cheng B., Zhang K. (2017). Pharmacist-driven antimicrobial stewardship in intensive care units in East China: a multicenter prospective cohort study. *American Journal of Infection Control*.

[60] Dalton K., Byrne S. (2017). Role of the pharmacist in reducing healthcare costs: current insights. *Integrated Pharmacy Research and Practice*.

[61] Nathwani D., Varghese D., Stephens J., Ansari W., Martin S., Charbonneau C. (2019). Value of hospital antimicrobial stewardship programs [ASPs]: a systematic review. *Antimicrobial Resistance and Infection Control*.

[62] Ashiru-Oredope D., Budd E. L., Bhattacharya A. (2016). Implementation of antimicrobial stewardship interventions recommended by national toolkits in primary and secondary healthcare sectors in England: TARGET and start smart then focus. *Journal of Antimicrobial Chemotherapy*.

[63] Russ S. J., Sevdalis N., Moorthy K. (2015). A qualitative evaluation of the barriers and facilitators toward implementation of the WHO surgical safety checklist across hospitals in England: lessons from the Surgical Checklist Implementation Project. *Annals of Surgery*.

[64] Colligan C., Sneddon J., Bayne G., Malcolm W., Walker G., Nathwani D. (2015). Six years of a national antimicrobial stewardship programme in UK: where are we now?. *Antimicrobial Resistance and Infection Control*.

[65] Cooke F. J., Matar R., Lawson W., Aliyu S. H., Holmes A. (2010). Comment on: antibiotic stewardship—more education and regulation not more availability?. *Journal of Antimicrobial Chemotherapy*.

[66] Dyar O. J., Tebano G., Pulcini C. (2017). Managing responsible antimicrobial use: perspectives across the healthcare system. *Clinical Microbiology and Infections*.

[67] Greene M. H., Nesbitt W. J., Nelson G. E. (2020). Antimicrobial stewardship staffing: how much is enough?. *Infection Control & Hospital Epidemiology*.

[68] Brink A. J., Messina A. P., Feldman C., Richards G. A., Van Den Bergh D., Alliance N. A. S. S. (2017). From guidelines to practice: a pharmacist-driven prospective audit and feedback improvement model for peri-operative antibiotic prophylaxis in 34 South African hospitals. *Journal of Antimicrobial Chemotherapy*.

[69] Abubakar U., Tangiisuran B. (2020). Nationwide survey of pharmacists’ involvement in antimicrobial stewardship programs in Nigerian tertiary hospitals. *Journal of Global Antimicrobial Resistance*.

[70] Pollack L. A., Van Santen K. L., Weiner L. M., Dudeck M. A., Edwards J. R., Srinivasan A. (2016). Antibiotic stewardship programs in US acute care hospitals: findings from the 2014 national healthcare safety network annual hospital survey. *Clinical Infectious Diseases*.

[71] Harris A. D., McGregor J. C., Perencevich E. N. (2006). The use and interpretation of quasi-experimental studies in medical informatics. *Journal of the American Medical Informatics Association*.

[72] Howlett B., Shelton T. G., Rogo E. (2020). *Evidence Based Practice for Health Professionals*.

[73] Curtis E., Drennan J. (2013). *Quantitative Health Research: Issues and Methods*.

